# RETRACTION: Kruppel‐Like Factor 4 Improves Obesity‐related Nephropathy Through Increasing Mitochondrial Biogenesis and Activities

**DOI:** 10.1111/jcmm.71314

**Published:** 2026-09-03

## Abstract

**RETRACTION**: JinL. , YeH. , PanM. , ChenY. , YeB. , ZhengY. , HuangW. , PanS. , ShiZ. , and ZhangJ. , “Kruppel‐Like Factor 4 Improves Obesity‐related Nephropathy Through Increasing Mitochondrial Biogenesis and Activities,” Journal of Cellular and Molecular Medicine 24, no. 2 (2019): 1200‐1207, 10.1111/jcmm.14628.31800161 PMC6991690

The above article, published online on 4 December 2019 in Wiley Online Library (wileyonlinelibrary.com), has been retracted by agreement between the journal Editor‐in‐Chief, Stefan N. Constantinescu; the Foundation for Cellular and Molecular Medicine; and John Wiley & Sons Ltd. The retraction has been agreed due to concerns raised by third parties. Specifically, image elements in Figure 3C were found to have been previously published by a different author group in a different scientific context. Further investigation by the publisher identified undeclared splicing sites in the IκB blot in Figure 2F and in the α‐porin blot in Figure 4c. Finally, the tubulin blot in Figure 4c was also found to have been previously published by a different author group in a different scientific context.

The first author stated that the overlap of data with a previous publication lacking shared authorship was due to data theft from their laboratory. However, no further clarification or supporting raw data was provided for the remaining concerns. As a result, the article has been retracted as the editors have lost confidence in the integrity and reliability of the whole body of data and consider the article's conclusions invalid.

**RETRACTION**: L. Jin , H. Ye , M. Pan , Y. Chen , B. Ye , Y. Zheng , W. Huang , S. Pan , Z. Shi , and J. Zhang , “Kruppel‐Like Factor 4 Improves Obesity‐related Nephropathy Through Increasing Mitochondrial Biogenesis and Activities,” Journal of Cellular and Molecular Medicine 24, no. 2 (2019): 1200‐1207, 10.1111/jcmm.14628.31800161 PMC6991690

The above article, published online on 4 December 2019 in Wiley Online Library (wileyonlinelibrary.com), has been retracted by agreement between the journal Editor‐in‐Chief, Stefan N. Constantinescu; the Foundation for Cellular and Molecular Medicine; and John Wiley & Sons Ltd. The retraction has been agreed due to concerns raised by third parties. Specifically, image elements in Figure 3C were found to have been previously published by a different author group in a different scientific context. Further investigation by the publisher identified undeclared splicing sites in the IκB blot in Figure 2F and in the α‐porin blot in Figure 4c. Finally, the tubulin blot in Figure 4c was also found to have been previously published by a different author group in a different scientific context.

The first author stated that the overlap of data with a previous publication lacking shared authorship was due to data theft from their laboratory. However, no further clarification or supporting raw data was provided for the remaining concerns. As a result, the article has been retracted as the editors have lost confidence in the integrity and reliability of the whole body of data and consider the article's conclusions invalid.

